# Supplementary material for: MetaRibo-Seq measures translation in microbiomes
Source: Nat Commun. 2020 Jun 29;11:3268. doi: 10.1038/s41467-020-17081-z (PMC7324362; doi:10.1038/s41467-020-17081-z)
Supplement: Supplementary file 10 — Supplementary Data 7 [file 41467_2020_17081_MOESM10_ESM.zip › File2/Confidence_High_Taxonomy/276615_out.krona.html]

Javascript must be enabled to view this page.

members
magnitude
magnitudeUnassigned
count
unassigned
taxon
rank

276615\_out

10

2
10
superkingdom

phylum
1239
10

class
186801
10

186802
10
order

10
186803
family

39491
10

SRS013158\_contig\_number\_contig-100\_1848.38065SRS017191\_contig\_number\_16381SRS023526\_contig\_number\_39402SRS043701\_contig\_number\_14646SRS043768\_contig\_number\_18652SRS052697\_contig\_number\_19711SRS057478\_contig\_number\_9289SRS077194\_contig\_number\_contig-100\_850.192870SRS142503\_contig\_number\_57214SRS142599\_contig\_number\_12018
species
